# Supplementary material for: Emergence and propagation of epistasis in metabolic networks
Source: eLife. 2021 Feb 2;10:e60200. doi: 10.7554/eLife.60200 (PMC7924954; doi:10.7554/eLife.60200)
Supplement: Supplementary file 2. [file elife-60200-supp2.pdf]

Definitions:

```
$Assumptions = w12 ≥ 0 && w13 ≥ 0 && w16 ≥ 0 && w24 ≥ 0 && w25 ≥ 0 &&
   w36 ≥ 0 && w45 ≥ 0 && u > 0 && v > 0 && w21 ≥ 0 && w31 ≥ 0 && w61 ≥ 0 &&
   w42 ≥ 0 && w52 ≥ 0 && w63 ≥ 0 && w54 ≥ 0 && K34 > 0 && K56 > 0 && K31 > 0 && K51 > 0
w = {{0, w12, w13, 0, 0, w16}, {w21, 0, 0, w24, w25, 0}, {w31, 0, 0, u, 0, w36},
   {0, w42, u / K34, 0, w45, 0}, {0, w52, 0, w54, 0, v}, {w61, 0, w63, 0, v / K56, 0}}
```

w12 ≥ 0 && w13 ≥ 0 && w16 ≥ 0 && w24 ≥ 0 && w25 ≥ 0 && w36 ≥ 0 &&
w45 ≥ 0 && u > 0 && v > 0 && w21 ≥ 0 && w31 ≥ 0 && w61 ≥ 0 && w42 ≥ 0 &&
w52 ≥ 0 && w63 ≥ 0 && w54 ≥ 0 && K34 > 0 && K56 > 0 && K31 > 0 && K51 > 0

$$\left\{\{0, w12, w13, 0, 0, w16\}, \{w21, 0, 0, w24, w25, 0\}, \{w31, 0, 0, u, 0, w36\}, \left\{0, w42, \frac{u}{K34}, 0, w45, 0\right\}, \{0, w52, 0, w54, 0, v\}, \left\{w61, 0, w63, 0, \frac{v}{K56}, 0\right\}\right\}$$

```
MatrixForm[w]
```

ixForm=

$$\begin{pmatrix} 0 & w12 & w13 & 0 & 0 & w16 \\ w21 & 0 & 0 & w24 & w25 & 0 \\ w31 & 0 & 0 & u & 0 & w36 \\ 0 & w42 & \frac{u}{K34} & 0 & w45 & 0 \\ 0 & w52 & 0 & w54 & 0 & v \\ w61 & 0 & w63 & 0 & \frac{v}{K56} & 0 \end{pmatrix}$$

Sum of rows

```
d = Table[Total[w[[i, All]]], {i, 6}]
d34 = Collect[Expand[d[[3]] * d[[4]] - w[[3, 4]] * w[[4, 3]]], u]
d56 = Collect[Expand[d[[5]] * d[[6]] - w[[5, 6]] * w[[6, 5]]], v]
```

$$\left\{w12 + w13 + w16, w21 + w24 + w25, u + w31 + w36, \frac{u}{K34} + w42 + w45, v + w52 + w54, \frac{v}{K56} + w61 + w63\right\}$$

$$w31\,w42 + w36\,w42 + w31\,w45 + w36\,w45 + u\left(\frac{w31}{K34} + \frac{w36}{K34} + w42 + w45\right)$$

$$w52\,w61 + w54\,w61 + w52\,w63 + w54\,w63 + v\left(\frac{w52}{K56} + \frac{w54}{K56} + w61 + w63\right)$$

Effective reaction rates after elimination of metabolites 5 and 6 (useful for easy differentiation w.r.to u), and the final effective reaction rate y of module μ

```
W = Table[w[[i, j]] +
     1/d56 (d[[5]] * w[[i, 6]] * w[[6, j]] + d[[6]] * w[[i, 5]] * w[[5, j]] + w[[i, 5]] *
        w[[5, 6]] * w[[6, j]] + w[[i, 6]] * w[[6, 5]] * w[[5, j]]), {i, 4}, {j, 4}];
W = UpperTriangularize[W, 1] + LowerTriangularize[W, -1] ;
ynum56 =
  W[[1, 3]] * W[[3, 2]] * Total[W[[4, All]]] + W[[1, 4]] * W[[4, 2]] * Total[W[[3, All]]] +
   W[[1, 3]] * W[[3, 4]] * W[[4, 2]] + W[[1, 4]] * W[[4, 3]] * W[[3, 2]];
ydenom56 = Total[W[[3, All]]] * Total[W[[4, All]]] - W[[3, 4]] * W[[4, 3]];
y  = W[[1, 2]] + ynum56/ydenom56;
```

Effective reaction rates after elimination of metabolites 3 and 4 (useful for easy differentiation w.r.to v)

```
V = Table[w[[i, j]] +
     1/d34 (d[[3]] * w[[i, 4]] * w[[4, j]] + d[[4]] * w[[i, 3]] * w[[3, j]] + w[[i, 3]] *
        w[[3, 4]] * w[[4, j]] + w[[i, 4]] * w[[4, 3]] * w[[3, j]]), {i, 6}, {j, 6}];
V = UpperTriangularize[V, 1] + LowerTriangularize[V, -1];
V[[All, 3]]  = 0;
V[[3, All]]  = 0;
V[[All, 4]]  = 0;
V[[4, All]]  = 0;
```

Sanity check that the denominator obtained after elimination of 5,6 first and 3,4 second is the same as the one obtained after elimination of 3,4 first and 5,6 second are the same (multiplied by d34 and d56, respectively, which makes them polynomial).

```
dyduDenom  = Total[W[[3, All]]] * Total[W[[4, All]]]  - W[[3, 4]] * W[[4, 3]];
dydvDenom  = Total[V[[5, All]]] * Total[V[[6, All]]] - V[[5, 6]] * V[[6, 5]];
Simplify[dydvDenom * d34]  == Simplify[dyduDenom * d56]
```

```
True
```

Defining numerators and denominators for the derivatives w.r.t to u and v

```
dyduNum = W[[3, 1]] * W[[4, 2]] - W[[3, 2]] * W[[4, 1]];
dydvNum  = V[[5, 2]] * V[[6, 1]] - V[[5, 1]] * V[[6, 2]];
```

Derivatives of y w.r.to u and v

$$dydu = \frac{1}{K31} * \left(\frac{dyduNum}{dyduDenom}\right)^2;$$

$$dydv = \frac{1}{K51} * \left(\frac{dydvNum}{dydvDenom}\right)^2;$$

Collecting coefficients in the numerators and denominators of the dydu derivative:

```
dyduNumCoefList = Map[Simplify, CoefficientList[dyduNum * d56, v]]
dyduDenomCoefList = Map[Simplify, CoefficientList[dyduDenom * d56, v]]
ψ  =  w42 * w52 + w54 * w42 + w45 * w52;
ϕ = w31 * w61  +  w63 * w31 + w36 * w61;
au  =  ψ * w31 / K56 + w42 *  ϕ;
bu  = ψ * ϕ;
eu = u * ((w31 * w52 + w31 * w54 + w36 * w52) / K34 / K56 + (w42 * w61 + w42 * w63 + w45 * w61)) +
    d[[4]] * ϕ + d[[3]] * ψ / K56;
fu = u * ((w52 + w54) / K34 * ϕ + (w61 + w63) * ψ) + ϕ * ψ
Expand[dyduNumCoefList[[1]]] == Expand[bu]
Expand[dyduNumCoefList[[2]]] == Expand[au]
Expand[dyduDenomCoefList[[1]]] == Expand[ fu]
Expand[dyduDenomCoefList[[2]]] == Expand[ eu]
```

$$\left\{(w45\,w52 + w42\,(w52 + w54))\,(w36\,w61 + w31\,(w61 + w63)),\ \frac{1}{K56}(w31\,w45\,w52 + K56\,w36\,w42\,w61 + w31\,w42\,(w52 + w54 + K56\,(w61 + w63)))\right\}$$

$$\left\{(w45\,w52 + w42\,(w52 + w54))\,(w36\,w61 + w31\,(w61 + w63)) + \frac{1}{K34}u\,(w36\,(w52 + w54)\,w61 + w31\,(w52 + w54)\,(w61 + w63) + K34\,(w45\,w52 + w42\,(w52 + w54))\,(w61 + w63)),\ \frac{1}{K34\,K56}(u\,(w36\,(w52 + K56\,w61) + K34\,w45\,(w52 + K56\,w61) + w31\,(w52 + w54 + K56\,(w61 + w63)) + K34\,w42\,(w52 + w54 + K56\,(w61 + w63))) + K34\,(w36\,w45\,(w52 + K56\,w61) + w36\,w42\,(w52 + w54 + K56\,w61) + w31\,w45\,(w52 + K56\,(w61 + w63)) + w31\,w42\,(w52 + w54 + K56\,(w61 + w63)))))\right\}$$

$$(w42\,w52 + w45\,w52 + w42\,w54)\,(w31\,w61 + w36\,w61 + w31\,w63) + u\left((w42\,w52 + w45\,w52 + w42\,w54)\,(w61 + w63) + \frac{(w52 + w54)\,(w31\,w61 + w36\,w61 + w31\,w63)}{K34}\right)$$

```
True

True

True

True
```

Collecting coefficients in the numerators and denominators of the dydv derivative:

```
dydvNumCoefList = Map[Simplify, CoefficientList[dydvNum * d34, u]]
dydvDenomCoefList = Map[Simplify, CoefficientList[dydvDenom * d34, u]]
av  =  ψ * w61 +  ϕ * w52 / K34;
bv  = ψ * ϕ;
ev = v * ((w31 * w52 + w31 * w54 + w36 * w52) / K34 / K56 + (w42 * w61 + w42 * w63 + w45 * w61)) +
    d[[5]] / K34 * ϕ + d[[6]] * ψ;
fv = v * ((w42 + w45) * ϕ + (w31 + w36) / K56 * ψ) + ϕ * ψ
Expand[dydvNumCoefList[[1]]] == Expand[bv]
Expand[dydvNumCoefList[[2]]] == Expand[av]
Expand[dydvDenomCoefList[[1]]] == Expand[ fv]
Expand[dydvDenomCoefList[[2]]] == Expand[ ev]
```

$$\left\{(w45\,w52 + w42\,(w52 + w54))\,(w36\,w61 + w31\,(w61 + w63)),\ \frac{w36\,w52\,w61 + K34\,(w45\,w52 + w42\,(w52 + w54))\,w61 + w31\,w52\,(w61 + w63)}{K34}\right\}$$

$$\left\{\frac{v\,(w31 + w36)\,(w45\,w52 + w42\,(w52 + w54))}{K56} + (v\,(w42 + w45) + w45\,w52 + w42\,(w52 + w54))\,(w36\,w61 + w31\,(w61 + w63)),\ \frac{1}{K34\,K56}(K56\,(w36\,(w52 + w54)\,w61 + w31\,(w52 + w54)\,(w61 + w63) + K34\,(w45\,w52 + w42\,(w52 + w54))\,(w61 + w63)) + v\,(w36\,(w52 + K56\,w61) + K34\,w45\,(w52 + K56\,w61) + w31\,(w52 + w54 + K56\,(w61 + w63)) + K34\,w42\,(w52 + w54 + K56\,(w61 + w63))))\right\}$$

$$(w42\,w52 + w45\,w52 + w42\,w54)\,(w31\,w61 + w36\,w61 + w31\,w63) + v\left(\frac{(w31 + w36)\,(w42\,w52 + w45\,w52 + w42\,w54)}{K56} + (w42 + w45)\,(w31\,w61 + w36\,w61 + w31\,w63)\right)$$

```
True

True

True

True
```

Obtaining the pre-factor β in the second derivative d2ydudv

```
d2ydudvNum1 = Simplify[dyduNumCoefList[[2]] * dyduDenomCoefList[[1]] -
     dyduNumCoefList[[1]] * dyduDenomCoefList[[2]]];
β = -w36 * ψ / K56 - w45 * ϕ;
Simplify[d2ydudvNum1 / (dydvNum * d34)] == Simplify[β]
True
```

Another way of obtaining β

```
Simplify[(D[dyduNum, v] * dyduDenom - dyduNum * D[dyduDenom, v]) *
   d56^2 / dydvNum / d34] == Simplify[β]
```

True

Verifying that β in the second derivative d2ydvdu is the same as above

```
d2ydvduNum1 = Simplify[dydvNumCoefList[[2]] * dydvDenomCoefList[[1]] -
    dydvNumCoefList[[1]] * dydvDenomCoefList[[2]]];
Simplify[d2ydvduNum1 / (dyduNum * d56)]
```

$$-\frac{w36\,w54\,w61 + K34\,(w45\,w52 + w42\,(w52 + w54))\,w63 + w31\,w54\,(w61 + w63)}{K34}$$
